# A Successful Way of Capping Nerves

**Published:** 1870-10

**Authors:** W. E. Driscoll

**Affiliations:** Bedford, Indiana


### A SUCCESSFUL WAY OF CAPPING NERVES.

BY W. E. DRISCOLL, BEDFORD, INDIANA.

It seems quite evident, from what we hear of the experiments made with oxychloride of zinc, for capping nerves or pulps, that the results are anything but uniform with the different operators.

Once it was the source of the greatest humiliation with me that I could not claim such success as others had. Now, it is my greatest pride that I *can.*

My present mode and the one with which I have had such gratifying success, is the same as Dr. W. H. Morgan describes in the August number of the REGISTER. I hesitated to intrude my views upon the notice of the profession, but backed by such authority that the plan is good, I hope the attention of all will be called to it again and again, until we see if it is not a mode by which all can be successful. Turn to Dr. Morgan's article, read it, and give the plan a trial.

---

PAIN AFTER OPERATIONS.—The *Lancet* says, the narcotic effects of chloroform or ether destroy pain during operations, but many patients suffer severely after the narcotism has gone off. To prevent this, Prof. Sedillot, of Strasburg, proposes to divide the tissues with the platinum wire brought to a white heat by the galvanic current. This division should be effected very slowly, so as to prevent hemorrhage. The author states that no pain is felt on awaking; that the wounds heal extremely well; and that hospital complications do not occur.

---

A correspondent of the *British Medical Journal* speaks of sulphate of potassa as "almost a specific for enlarged tonsils in children, the result of debility." It is given daily for a month or six weeks in doses of "from five to fifteen grains every morning, with a small quantity of rhubarb and an aromatic." Mere laxness of the bowels should be produced, and the dose is to be at once diminished if purging occur.

